# Impact of the two-dose rubella vaccination regimen on incidence of rubella seronegativity in gravidae aged 25 years and younger

**DOI:** 10.1371/journal.pone.0183630

**Published:** 2017-08-30

**Authors:** Shuk Yi Annie Hui, Daljit S. Sahota, Terence T. Lao

**Affiliations:** Department of Obstetrics & Gynaecology, The Chinese University of Hong Kong, Hong Kong, People’s Republic of China; Public Health England, UNITED KINGDOM

## Abstract

**Objective:**

This study compared the incidence of rubella seronegativity among gravidae of 25 year-old and younger, between those born in Hong Kong after 1983 when the two-dose rubella vaccination was implemented, versus gravidae born before, to examine the impact of the two-dose regimen.

**Methods:**

In this retrospective cohort study, the incidence of antenatal rubella seronegativity in our parturients managed in1997-2015 was analysed by their age from ≤16 to 25 years, and the effect of year of birth was determined adjusting for confounding factors including teenage status, obstetric history, anthropometric factors, and health parameters including anaemia, thalassaemia trait and hepatitis B carrier status.

**Results:**

Among the 12743 gravidae, the 6103 gravidae born after 1983 had overall higher rubella seronegativity (9.1% versus 4.4%, OR 2.061, 95% CI 1.797–2.364), with significant difference (p = 0.006) and inverse correlation (p<0.001) with age, in contrast to the 6640 gravidae born in/before 1983 whom there was significant difference (p = 0.027) but a positive correlation (p = 0.008) with age. For each year of age, the former had significantly higher incidence of rubella seronegativity except for those of ≤16 years. Regression analysis confirmed that birth after 1983 was independently associated with rubella seronegativity (aOR 2.207, 95% CI 1.902–2.562).

**Conclusion:**

There was a significant trend between rubella seronegativity with age in young gravidae, but the pattern was opposite between gravidae born after versus in/before 1983, with the former having a higher incidence of seronegativity at all ages. Young women covered by the two-dose rubella immunisation programme have a paradoxically higher incidence of rubella seronegativity.

## Introduction

The successful implementation of the highly effective mumps-measles-rubella (MMR) vaccine can be best exemplified by the experience in Finland, where the 2-dose schedule introduced since 1982 reduced all MMR diseases to 0.1 per 100,000 population in 1995 [1]. A more recent example is Spain, where a 54% increase in MMR vaccination coverage from 2003 (one dose) to 2013 (two doses) resulted in 95.5% of the mothers and 96.4% of their neonates to having protective levels of anti-rubella IgG [2]. Yet ironically, there are emerging reports of declining rubella immunity and decreasing incidence of protective antibody levels (≥10 IU/ml) among women born after the introduction of routine universal rubella immunisation in both European and Asian countries [3–11]. Indeed, national figures in Ireland showed that rubella seronegativity was 14.7% among gravidae <25 years old compared to that of 5.0% in older gravidae [11]. The observations above led to the concern that the MMR programme may only be effective at preventing childhood rubella [6].

In Hong Kong, mandatory one-dose rubella immunisation for schoolgirls, postpartum mothers, and susceptible women of child-bearing age was first introduced in 1978 [12], and extended to cover to all infants at 12 months of age from 1990, with documented coverage of over 98% for primary six schoolgirls [12,13]. Free vaccination to citizens and their offspring is also provided by the Department of Health (DH) where necessary [12]. From September 1996, a second dose of MMR vaccine was introduced to all primary six students to replace the rubella vaccine which was previously given to school girls only. The age of eligibility for this second dose was lowered and brought forward to primary one students from June 1997. Mop-up exercises were further conducted annually in primary six to ensure adequate immunization of students against measles. This two dose regime was documented with 99% coverage of all primary six students [13,14], and which would have covered all children born after 1983. The children’s immunisation record would be checked at primary schools for any necessary remedial action [12,14]. These measures most likely explained the small number of reported rubella cases of between 19 and 53 from 2001 to 2008, and only one case of congenital rubella syndrome (CRS) in 2008 [15,16].

Nevertheless, Asians and Chinese appear to be particularly prone to have an overall lower rate of seropositivity or protective antibody titre as compared to Caucasians in multiethnic communities [17–20]. We have previously found that the incidence of rubella seronegativity had decreased from 12.6% in gravidae born in Hong Kong before 1965 to 6.5% in those born after 1983, when each individual would have received two doses at the age of 12 months and 12 years [21]. However, similar to the finding in Ireland, older adolescents and young adults in China were recently found to have lower rubella seroprevalence [22]. Seroprevalence was lower in female than in male adults, and 50.54% of rubella cases were observed in subjects aged 15–39 years. As most adolescent women will eventually become mothers, there could emerge an unanticipated increase in the incidence of CRS consequent to a hitherto unknown escalating proportion of rubella seronegativity among female vaccinees. This study was therefore conducted to clarify the relationship between age and rubella seronegativity. Among the Hong-Kong-born gravidae aged 25 years or younger, we compared those born after 1983 versus those born in/before, to determine the impact of the two-doses regimen on the age-specific incidence of seronegativity.

## Material and methods

In Hong Kong, routine antenatal screening for rubella IgG antibody by EIA (Axsym, Abbott) is performed by the accredited central laboratory under DH and accredited hospital pathology laboratories. Gravidae with a titre of <10 IU/ml are considered seronegative and will be referred for postpartum rubella vaccination. In addition, there is routine screening for hepatitis B surface antigen due to the high local prevalence of hepatitis B virus (HBV) infection and carrier status, to ensure that infants of HBV carrier mothers are given timely passive-active immunisation after birth [23]. Results of the antenatal screening and clinical information are captured in a computerised database set up by the local Hospital Authority for the generation of statistics. Data entry is made by trained midwives and obstetricians in the clinics and wards and is double-checked after delivery.

Our hospital is a regional referral centre catering for 1.7 million people. For this retrospective cohort study approved by the Institutional Review Board (IRB) of The Joint Chinese University of Hong Kong–New Territories East Cluster Clinical Research Ethics Committee (CRE-2009.271), all the data were fully anonymized before access by the researchers, and the IRB has waived the requirement for patient informed consent. We selected pregnant women aged ≤25 years, who were born in Hong Kong and managed from January 1997 to December 2015. This study included all the eligible subjects in the database who had received our care, with data on the required information for analysis, irrespective of the pregnancy outcome (miscarriage, termination, or delivery) or the eventual place of delivery (in our hospital or other hospitals). The cases for this study were extracted with the relevant information from the master database to create a research database (for anonymity) for analysis.

Maternal demographic and anthropometric data, obstetric and medical history, and result of rubella and HBV screening, and were extracted from our hospital database, which was validated previously [23]. We labelled women with rubella-specific IgG level <10 IU/mL as having rubella seronegative status as it was impossible to establish whether their low antibody titre was due to primary or secondary vaccine failure, or whether they were naïve to the natural infection or vaccination. Maternal characteristics including age, parity, height, weight, body mass index (BMI in kg/m^2^, calculated using weight and height measured at antenatal booking), year of birth categorised into after 1983 (actual year of birth 1984–1998) or in/before (actual year of birth 1972–1983), presence of iron deficiency anaemia as surrogate for nutritional status, presence of any thalassaemia trait, and HBV status, were retrieved for analysis. We examined the impact of both high (≥25 kg/m^2^) [24] and low (<18.5 kg/m^2^) BMI [25], as well as short stature (< 151 cm) as a surrogate of nutritional status during childhood and adolescence. Thalassaemia traits are common in our locality [26], but its relationship with immune response to the MMR vaccine has not been examined so that this was examined to elucidate its role in the high incidence of rubella seronegativity. We examined the impact of chronic HBV infection as we have found an association between rubella seronegativity with chronic HBV infection before [23]. Gravidae born after 1983 were compared with those born in/before, and then gravidae at each age were examined in turn.

For statistical analysis, the Student’s t-test was used for continuous variables and chi-square test and Spearman’s correlation for categorical variables. Comparison between gravidae born after versus in/before 1983 was performed using chi-square test, odds ratio (OR) was calculated with 95% confidence intervals (CI), and the trend with year-of-birth was tested with Spearman’s correlation. Multiple logistic regression analysis was performed to determine the independent effect of teenage and year of birth after 1983, adjusting for the potential confounding variables examined in the univariate analysis, yielding the adjusted odds ratio (aOR) with 95% CI. Different models were used to determine the influence of confounding variables on the association, if any, between teenage and the year of birth with rubella seronegativity. Statistical analysis was performed using a commercially available statistical package (IBM SPSS Statistics version 20).

## Results

There were 12743 gravidae eligible for the study, accounting for 96.0% of the 13276 cases aged ≤25 years in the database. Gravidae born after 1983 showed comparable mean age, despite a higher incidence of teenage (≤19 years). This group also showed no difference in mean weight, but a higher mean BMI, which was probably related to the significantly shorter mean height. For obstetric history, those born after 1983 had more primigravidae and nulliparae, and fewer previous abortions. For their health status, there was no difference in the incidence of iron deficiency anaemia, but the significantly higher incidence of thalassaemia traits and other medical conditions. The rubella seronegativity (9.1% versus 4.4%, p<0.001, OR 2.061, 95% CI 1.797–2.364) was also significantly higher, even though the incidence of HBV infection was lower. (Table 1)

**Table 1:** Maternal characteristics of gradivae 25 years and younger born > 1983 versus ≤ 1983.

	Year-of-birth			
	>1983	≤1983	*P*	OR (95% CI)
Number (% of total)	6103	6640	-	-
Age (years)	21.4±2.0	21.6±2.9	0.198	-
Teenager (%)*	15.3	10.0	<0.001	1.525 (1.389–1.674)
Weight (kg)	55.2±10.1	54.8±9.0	0.349	-
Height (cm)	157.8±5.7	158.5±5.6	<0.001	-
% <151cm*	7.6	7.9	0.529	0.962 (0.852–1.086)
BMI (kg/m^2^)	22.1±3.7	21.8±3.3	0.019	-
% ≥25.0 kg/m^2^ *	14.8	14.9	0.856	0.992 (0.913–1.078)
% <18.5 kg/m^2^ *	14.0	11.5	<0.001	1.221 (1.113–1.339)
Primigravidae (%) *	49.7	43.4	<0.001	1.147 (1.105–1.190)
Prior abortions (%) *	38.0	44.1	<0.001	0.861 (0.826–0.898)
Nulliparae (%) *	78.0	76.2	0.009	1.026 (1.006–1.045)
Iron deficiency anaemia (%) *	1.4	1.1	0.126	1.273 (0.934–1.736)
Thalassaemia trait (%) *	6.6	4.7	<0.001	1.402 (1.189–1.652)
HBsAg seropositive (%) *	4.9	8.4	<0.001	0.582 (0.508–0.667)
Rubella seronegative (%) *	9.1	4.4	<0.001	2.061 (1.797–2.364)
Medical conditions (%) *	13.8	11.7	<0.001	1.180 (1.078–1.293)

Results expressed in mean±standard deviation (SD) and analysed by t test, or in % and analysed by chi square test as indicated by *.

**Fig 1:**
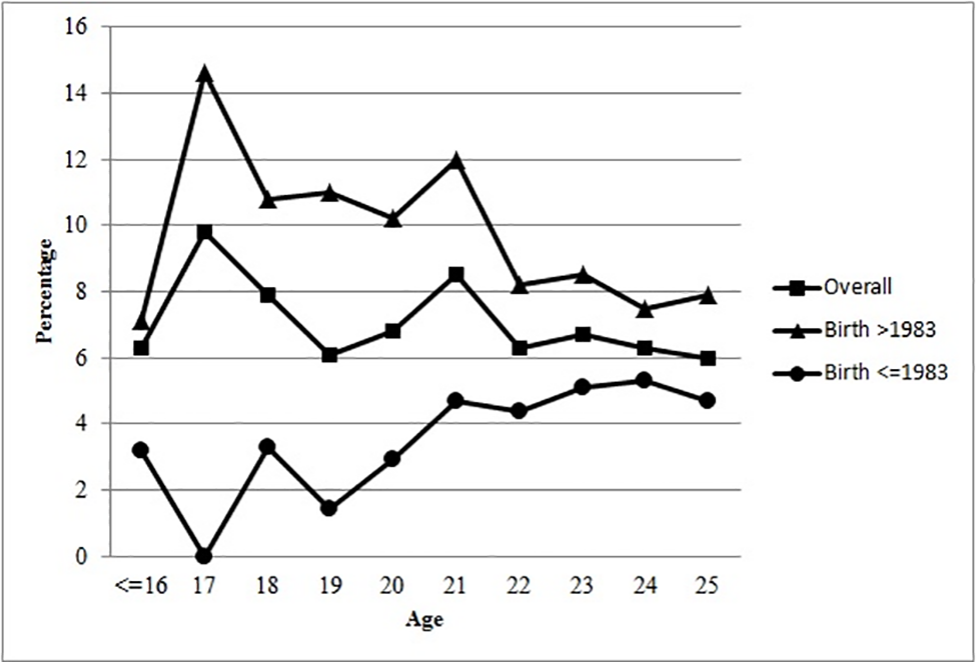
Incidence of rubella seronegativity in Hong Kong-born gravidae aged ≤25, comparing birth >1983 versus ≤1983. ■ Overall group all gravidae: difference among groups p = 0.084, trend with age p = 0.016. ▲Gravidae born after 1983: difference among groups p = 0.006; trend with age p<0.001. • Gravidae born in/before 1983: difference among groups p = 0.027, trend with age p = 0.008

When the overall incidence of rubella seronegativity was related with maternal age, the difference among the different age groups failed to reach statistical significance (p = 0.084) although there was a slightly but significantly decreasing trend (p = 0.016). However, when stratified by birth after 1983 versus in/before, the former group showed a significant difference (p = 0.006) and inverse correlation (p<0.001) with age, in contrast to the latter group in whom there was similarly significant difference (p = 0.027) but a positive correlation (p = 0.008) with age. (Fig 1) For gravidae born after 1983, all individual age group had significantly higher incidence of rubella seronegativity, except those of ≤16 years owing to the relatively small number of subjects. (Table 2).

**Table 2:** Maternal rubella seronegativity by age group, comparing between gravidae born > 1983 versus ≤ 1983.

Maternal age (years)	Year-of-birth cohort			
	>1983	≤1983	*P*	OR (95% CI)
≤16 (n = 143)	7.1 (8/112)	3.2 (1/31)	0.684*	2.214 (0.288–17.037)
17 (n = 254)	14.6 (25/171)	0.0 (0/83)	<0.001*	-
18 (n = 478)	10.8 (32/295)	3.3 (6/183)	0.003	3.308 (1.411–7.758)
19 (n = 722)	11.0 (39/354)	1.4 (5/368)	<0.001	8.108 (3.233–20.337)
20 (n = 1004)	10.2 (54/529)	2.9 (14/475)	<0.001	3.463 (1.950–6.153)
21 (n = 1230)	12.0 (76/633)	4.7 (28/597)	<0.001	2.560 (1.684–3.891)
22 (n = 1638)	8.2 (68/826)	4.4 (36/812)	0.002	1.857 (1.254–2.749)
23 (n = 2006)	8.5 (80/943)	5.1 (54/1063)	0.002	1.670 (1.196–2.332)
24 (n = 2384)	7.5 (81/1075)	5.3 (69/1309)	0.024	1.429 (1.048–1.950)
25 (n = 2884)	7.9 (93/1165)	4.7 (80/1719)	<0.001	1.697 (1.269–2.268)
Difference	0.006	0.027	-	-
Trend	<0.001	0.008	-	-

Results expressed in % (numbers). Difference analysed by chi square test (or Fisher’s test two tailed as indicated*) and trend analysed by Spearman’s correlation with p value shown.

Multiple logistic regression analysis was performed adjusting for the confounding effects of obstetric history, anthropometric factors, and health conditions (Table 3).

**Table 3:** Multivariate analysis on the association between teenage and birth after 1983 with rubella seronegativity.

	Model 1	Model 2	Model 3
Primigravidae	1.270 (0.782–2.062)	1.307 (0.917–1.863)	-
Prior abortion ≥1	0.910 (0.590–1.404)	1.024 (0.750–1.399)	-
Nuilliparae	1.105 (0.764–1.601)	0.963 (0.736–1.259)	-
Height <151cm	**1.449 (1.066–1.972)**	1.201 (0.935–1.543)	1.202 (0.936–1.543)
BMI ≥25 kg/m^2^	**1.308 (1.016–1.684)**	**1.264 (1.045–1.529)**	**1.247 (1.032–1.508)**
BMI <18.5 kg/m^2^	1.063 (0.804–1.407)	1.031 (0.832–1.277)	1.041 (0.840–1.289)
HBV carrier status	1.329 (0.963–1.833)	1.172 (0.885–1.553)	1.171 (0.884–1.551)
Iron deficiency anemia	1.515 (0.673–3.413)	-	-
Thalassemia traits	1.051 (0.548–2.016)	-	-
Medical conditions	0.811 (0.531–1.240)	0.979 (0.793–1.29)	-
Teenage gravidae	0.878 (0.664–1.162)	1.001 (0.809–1.237)	1.040 (0.843–1.283)
Year of birth >1983	**1.424 (1.176–1.724)**	**2.183 (1.881–2.535)**	**2.207 (1.902–2.562)**

Results expressed in aOR (95% CI), significant results shown in bold.

Model 1 –Including all potential confounding variables assessed in the univariate analysis Model 2 –Excluding iron deficiency anaemia and thalassaemia traits as confounding variables

Model 3 –Including only maternal anthropometric parameters, teenage, and HBV carrier status as confounding variables

When all factors were taken into consideration (Model 1), short stature, high BMI and birth after 1983 were independently associated with rubella seronegativity. Removing iron deficiency anaemia and thalassaemia traits (Model 2), the association with height becomes insignificant, but the aOR for birth after 1983 was increased from 1.424 (1.176–1.724) to 2.183 (1.881–2.535). When the analysis was repeated with anthropometric factors and HBV carrier status alone (Model 3), there was minimal change in the aOR to 2.207 (1.902–2.562) for birth after 1983, with the *Constant* at p<0.001 and *Exp(B)* at 5.655. Teenage per se was not associated with rubella seronegativity across all models, the only other factor that showed consistent association was high BMI.

## Discussion

In this study, we found among the gravidae aged 25 years or younger, those born after 1983 whom would be covered by the two-dose MMR vaccination programme introduced in 1996 had paradoxically a two-fold higher incidence of rubella seronegativity compared with gravidae born earlier. In fact, the higher incidence of rubella seronegativity was found consistently across the entire group from those aged ≤16 years up to aged 25 years, although the difference in the youngest group (≤16 years) did not reach statistical significance owing to the small number of gravidae.

Moreover, there was an opposite trend in the incidence of rubella seronegativity in the gravidae born in these two periods (>1983 versus ≤1983). (Fig 1) Those born after 1983 had significantly decreasing trend of rubella seronegativity with increasing age, while the opposite was seen in those born in/before 1983, converging towards age 25, overall the trend of the cohort appeared relatively stable. Without the stratified analysis, the impact of birth after 1983 would have been masked. Regression analysis confirmed that birth after 1983 was independently associated with more than two-fold increase in rubella seronegativity in gravidae aged ≤25 years, and high BMI was identified as the only other consistent risk factor in our analysis. Although the maximum incidence of rubella seronegativity was found in the teenage gravidae, being a teenager per se did not appear to have contributed to rubella seronegativity in the regression analysis.

The rubella component of the MMR vaccine is regarded as the most effective [27,28], yet antibody levels would still decline substantially over time even after two doses [27]. In the UK, MMR was given to children aged 12–15 months from 1988, followed by a measles-rubella immunisation campaign in 1994 for all 5–16 year-old, and finally a second dose of MMR for children aged 3–5 years since 1996. However when the birth cohort of 1976–80 was used as the reference, the rate ratios of anti-rubella IgG <10 IU/ml increased from 1.49, 6.06, to 29.34 for the cohorts of 1981–85, 1986–90, and >1990 respectively, and 14% of those born after 1990 had non-protective antibody levels [5]. Another study similarly found that antibody <10 IU/ml increased significantly from 3.8% in 2005 to 5.1% in 2009 among pregnant women, and which was 2.2% for those born before versus 14.0% in those born after 1983 [4]. These observations were supported by yet another study in England in which rubella non-immune pregnant women increased from 1.4% in 2004 to 6.9% in 2011, and from 1.6% in women born before 1976 to 17.8% among those born since 1986 [6]. Thus the pattern of increasing rubella seronegativity among subjects covered by universal immunisation as reported in the U.K., and found similarly in Sweden [3], Israel [7,8], Poland [9], Taiwan [10], Ireland [11], and China [22], is also demonstrated in our obstetric population. The problem may lie with the MMR vaccine, rather than the rubella component alone, as measles outbreaks have reappeared in developed countries where there were no problems of vaccine access, public health infrastructure, or health literacy, that 8.9% of immunised healthy children lacked protective antibody levels after a mean of 7.4 years [29]. Indeed, it was shown that 30% to 100% of all reported measles cases occurred in previously immunised students [30]. The increasing failure to sustain seroprevalence to two of the three components of the MMR vaccine in children and adolescents who had received universal immunisation in childhood suggests that there is room for improvement in the current MMR immunisation protocol, for which further studies are warranted.

The underlying explanation of our observation remains to be determined. In our young gravidae, neither maternal anthropometric factors except for a high BMI nor past medical history or health status, appeared to have played any role. Even the presence of chronic HBV infection that has been associated with rubella seronegativity in our general obstetric population [23] was excluded as a significant factor. Immune response to childhood rubella vaccination was shown to be influenced by age at vaccination, as rubella seropositivity was highest in children aged 6–11 years (96.2%), followed by children aged 12–19 years (93.7%), and lowest in adult women (88.9% to 91.5%) in the US where vaccination coverage is >90% [31]. Ageing is known to impair T-cell function [32–34]. However, late age at immunisation and significant ageing are unlikely to have played any role in gravidae aged 25 years and younger. As high BMI was shown to be independently associated with rubella seronegativity in our gravidae, further studies are warranted to elucidate the relationship between maternal BMI and rubella seronegativity.

For the opposite trends in the relationship between rubella seronegativity with age between our gravidae born after versus in/before 1983, we have come up with a hypothesis to explain ours as well as the findings reported elsewhere. Our young gravidae born in/before 1983 were covered by catch-up programmes, therefore probably had received additional MMR immunisation some time in later childhood [12,13], albeit at different ages and assumingly much later than the standard dose at 12 months of age, with variable intervals from the index pregnancy. Hence the older they were at antenatal booking, the higher would be the incidence of rubella seronegativity as antibody levels naturally decline over time [27]. On the other hand, gravidae born after 1983 would all have received the two doses of MMR vaccine by the age of 12 years. They would have grown up among peers who were similarly immunised, so that their chance of exposure to the natural infection from the wild-type virus which could serve as mini-boosters to maintain immunity would have been minimal or even non-existent. However, with the coming of age, their increasing exposure to older often non-immune subjects increased their chance of exposure to subclinical rubella infection that could serve as natural boosters to enhance their immunity, particularly among those with an adequate anamnestic response. Thus the incidence of rubella seronegativity decreased progressively with age. Eventually rubella seronegativity would plateau at a constant level determined by the interactions of genetic factors, various doses of catch-up and postpartum vaccination and exposure to natural infection, for each successive generation within a specific community.

In Hong Kong, congenital cataract is routinely screened by red reflex examination for all newborns before they are discharged from the hospital. Although there was no systematic data showing the incidence across the years, a local study was conducted by the Hong Kong Eye Hospital and the School of Visually Impaired in 2005, looking at the causes of childhood blindness of 82 students, none was related to rubella infection.[35] This indirect evidence suggests that the MMR vaccination programme is highly effective. Although rubella IgG antibodies >10 IU/ml are commonly used as the cut off for immunity, this concentrations will decline over time; yet immunological memory seems to persist even with very low levels of IgG, and a secondary immune response (anamnestic response) will occur upon new exposure to rubella virus. While concerns have been raised whether the sensitivity could be increased by considering equivocal results as positive or reappraise the 10 IU/ml cut off,[36] for the purpose of our report we have to continue with our current definition which was based on the qualitative report issued by our public laboratories and used throughout the study period. Limited by the absence of data on rubella antibody titre, our finding of rubella seronegativity may not equate susceptibility since the proportion of women with intact anamnestic response remained unknown. Nevertheless, the opposite trends in the association between rubella seronegativity with age for gravidae born after 1983 versus those born earlier suggested strongly that different mechanisms operated in these two groups to produce these distinct patterns. While the association with BMI probably reflected the effect of obesity on immune response, that with year of birth most likely reflected the influence of a number of undetermined factors for which year of birth only served as proxy. Our observation is compatible with that in the UK and elsewhere where marked increases in rubella seronegativity were found following the introduction of immunisation [3–11]. Although antenatal screening for rubella immunity may have no advantage for the current pregnancy but served instead to increase the workload of laboratory and maternity units [6], in many places, this practice may be the only feasible and reliable means of monitoring rubella immunity in the population which clinicians are most concerned about. Meanwhile, further studies are warranted to explore remedial measures such as regular boosters in late adolescence or early adulthood in order to maintain rubella immunity in women of reproductive age.

## Conclusions

Antenatal rubella seronegativity among young gravide aged ≤25 years was paradoxically increased among those born after the introduction of the two-dose MMR immunisation regimen, compared with gravidae of the same age groups who were born before this regimen, therefore rubella immunity cannot be assumed in gravidae who have received the complete two-dose MMR coverage. The necessity of remedial measures, such as regular boosters from late adolescence, should be examined in future studies to optimise the long-term protection of fertile women against rubella infection.
